# Advances in Oral Biomacromolecule Therapies for Metabolic Diseases

**DOI:** 10.3390/pharmaceutics17020238

**Published:** 2025-02-12

**Authors:** Qiuxia Jiao, Yuan Huang, Jinhan He, Yining Xu

**Affiliations:** 1 Department of Pharmacy, Institute of Metabolic Diseases and Pharmacotherapy, West China Hospital, Sichuan University, Chengdu 610041, China; 2 Laboratory of Drug-Targeting and Drug Delivery System of the Education Ministry, West China School of Pharmacy, Sichuan University, Chengdu 610041, China

**Keywords:** oral biomacromolecules, metabolic diseases, oral drug delivery, gastrointestinal tract

## Abstract

Metabolic diseases like obesity and diabetes are on the rise, and therapies with biomacromolecules (such as proteins, peptides, antibodies, and oligonucleotides) play a crucial role in their treatment. However, these drugs are traditionally injected. For patients with chronic diseases (e.g., metabolic diseases), long-term injections are accompanied by inconvenience and low compliance. Oral administration is preferred, but the delivery of biomacromolecules is challenging due to gastrointestinal barriers. In this article, we introduce the available biomacromolecule drugs for the treatment of metabolic diseases. The gastrointestinal barriers to oral drug delivery and strategies to overcome these barriers are also explored. We then discuss strategies for alleviating metabolic defects, including glucose metabolism, lipid metabolism, and energy metabolism, with oral biomacromolecules such as insulin, glucagon-like peptide-1 receptor agonists, proprotein convertase subtilisin/kexin type 9 inhibitors, fibroblast growth factor 21 analogues, and peptide YY analogues.

## 1. Introduction

Metabolic diseases include obesity, diabetes, metabolic dysfunctions associated with steatosis liver disease (MASLD), cardiovascular disease (CVD), etc. [1]. Their main metabolic defects include dysglycemia, dyslipidemia, and lipid accumulation [2,3,4]. These pathological features usually influence and reinforce each other. According to the latest statistics from the World Health Organization, the prevalence of all metabolic diseases increased from 2000 to 2019 [5]. For example, more than a billion individuals currently have risk factors associated with obesity [6]. Furthermore, metabolic diseases are often associated with multiple complications, further aggravating medical costs and increasing mortality [7,8,9,10,11]. Among them, the largest number of deaths from metabolic diseases is due to obesity, with more than 5 million deaths per year up to 2019 [5]. The World Obesity Federation predicts that the global economic impact of overweight and obesity will reach USD 4.32 trillion annually by 2035 [12].

Biomacromolecule therapeutics, such as proteins, peptides, antibodies, and oligonucleotides, play a key role in the treatment of metabolic diseases, such as obesity and diabetes [13,14,15,16,17,18,19,20]. For example, insulin, a protein drug that regulates the body’s blood glucose levels, has been in clinical use for more than 100 years and helps diabetic patients [21]. Glucagon-like peptide-1 receptor agonists (GLP-1RAs), such as semaglutide, liraglutide, and exenatide, are an appealing option for the treatment of type 2 diabetes mellitus (T2DM) and obesity [22,23]. Peptide GLP-1RAs have been shown to be effective in reducing hemoglobin A1c (HbA1c) and body weight with a low risk of hypoglycemia [24]. It is worth noting that semaglutide was also approved by the United States Food and Drug Administration (FDA) for the treatment of cardiovascular disease in March 2024 [25]. Furthermore, this class of drugs has also been shown to improve liver steatosis and neurological diseases [26,27,28]. Proprotein convertase subtilisin/kexin type 9 (PCSK9) antibodies and small interfering RNA (siRNA) are major breakthroughs in cardiovascular therapeutics that have demonstrated unparalleled efficacy in alleviating hypercholesterolemia and reducing cardiovascular risk [29,30,31]. However, almost all biomacromolecule drugs must be administered by injection, which causes great inconvenience to patients. Long-term injections may cause inflammation and fibrosis at the injection site and affect the absorption and utilization of the administered substance. The inconvenience associated with injections reduces patient compliance [32,33,34].

Oral administration is one of the most preferred administration methods for patients. The non-invasive nature of oral administration mitigates complications linked to invasive routes, including pain, anxiety, and the risk of infection [35,36,37]. In addition to enhancing patient compliance and safety, oral administration could also offer the benefit of reduced healthcare costs [38,39]. Furthermore, for some biomacromolecules, oral delivery can provide additional compensation for disease treatments. For example, oral GLP-1 mimics can simulate natural GLP-1 secretion from the gut and retain its physiological benefits [24,40]. When drugs are effective due to high physiologic accumulation in the liver or specific action on the liver (e.g., insulin and fibroblast growth factor 21 (FGF21)) [38,41,42], these drugs can passively achieve liver-targeting due to the first-pass effect after oral administration, reducing physiologic accumulation in non-target tissues. However, there are currently only very few oral biologics available in clinics. Oral semaglutide tablets (Rybelsus) were approved by the FDA in 2019 for T2DM treatment, marking a milestone in the oral delivery of biomacromolecules over the last century [43,44]. Despite this, Rybelsus still has many limitations, such as strict fasting restrictions and low bioavailability (less than 1%) [45,46,47]. The poor oral bioavailability of biomacromolecules is due to the physiological and biological barriers in the gastrointestinal tract (GIT), such as the acidic environment in the stomach, various digestive enzymes, and poor epithelial permeability [48,49,50,51,52].

Recent advancements in biotechnology and nanotechnology in oral delivery are focused on overcoming these obstacles, with an emphasis on enhancing stability and improving the intestinal absorption of these biomacromolecules [53,54]. A variety of oral delivery strategies are exploited, such as penetration enhancers (PEs), nanoparticulate systems, and oral microrobots [32,53,55,56,57,58], which bring great improvements to this field. While existing reviews broadly cover topics such as oral delivery strategies (bioavailability enhancement) [32,34,35,48,50,53,55,57] and their use in specific biomacromolecules such as insulin [32,34,38,43,44,45,46,54,56], this article reviews a distinct perspective by focusing on the clinical application of biomacromolecules in the treatment of metabolic diseases. Specifically, we discuss the absorption barriers and advanced technologies for oral delivery, as well as the therapeutic potential of these strategies in addressing multiple metabolic disorders.

## 2. Biomacromolecular Drugs for Metabolic Diseases

In 2023, seven of the top ten best-selling drugs in the world were biomacromolecular drugs. It has been predicted that by 2025, four biological drugs for metabolic diseases will appear in the top ten best-selling drugs list [59]. These four are peptide drugs based on GLP-1, used to treat diabetes and obesity. Semaglutide currently ranks second amongst the top-selling medicines, including three brands, namely Ozempic hypoglycemic injections, Rybelsus hypoglycemic tablets, and Wegovy weight loss injections [60]. Semaglutide sales in 2023 reached USD 38.6 billion, nearly double its sales in 2022 [60]. Which continuously increased in the first half of 2024, reaching USD 13 billion [61]. In March 2024, Wegovy also received an additional label for contributing to cardiovascular risk reduction [62]. Tirzepatide, a GLP-1/glucose-dependent insulinotropic polypeptide (GIP) receptors co-agonist, was first approved for diabetes treatment in May 2022 [63] and for weight loss in November 2023 [64]. Mounjaro (hypoglycemic injection) and Zepbound (weight loss injection) had combined sales of USD 6.658 billion in the first half of 2024, up 330% year on year [65], becoming the fourth best-selling drugs in the world and the fastest-growing drugs of all time. Insulin was introduced in 1921 [34,66], and its annual sales are also steadily increasing due to an increased prevalence of diabetes and obesity, reaching 10% [67]. It is expected that by 2025, the global insulin market will reach approximately USD 43.3 billion [68]. PCSK9 drugs, such as PCSK9 antibodies and siRNA therapies, are novel therapeutics for metabolic diseases [69,70]. The FDA has approved these PCSK9 biologics for patients who are unable to achieve adequate low-density lipoprotein cholesterol (LDL-C) control with statins alone, which shows significant market potential [71,72]. For example, a PCSK9-targeting siRNA therapy, inclisiran (Leqvio), is expected to experience an impressive compound annual growth rate (CAGR) of 31.3% in its sales from 2024 to 2034 [73]. Nevertheless, many biomacromolecular drugs for metabolic diseases, such as Wegovy, Zepbound, and Leqvio, are still often in short supply, resulting in their high demand on the market.

Table 1 summarizes the available drugs on the market for the treatment of metabolic diseases (Table 1) [72,74,75,76,77,78]. With the exception of Rybelsus, almost all biomacromolecule therapies for metabolic diseases are injected. Additionally, only Leqvio could achieve the tissue-specific targeted delivery among these drugs. These further fuel the enthusiasm of the industry for developing advanced delivery strategies for these biomacromolecule drugs, such as oral administration. In this review, we focus on the advances of protein drugs, peptide drugs, monoclonal antibody drugs, and nucleic acid drugs in the treatment of metabolic diseases by oral delivery.

## 3. Oral Delivery of Biomacromolecular Drugs

Since oral biomacromolecular drugs are easy for patients to use, reduce costs, and increase comfort by avoiding the pain, anxiety, and risk of infection associated with injections [38,111,112], long-term medication adherence in patients with chronic metabolic diseases is higher [112,113,114,115,116]. In addition, oral administration does not require an auxiliary medical device to support its advantages in long-term treatment [38,117,118]. For some biological macromolecule drugs especially, such as insulin and peptide GLP-1 analogues, the oral route also has the advantage of simulating endogenous physiological secretion pathway, which is closer to the natural metabolic process of the human body and helps reduce the adverse reactions caused by the high concentration of drugs in non-target tissues [112,119]. However, oral biomacromolecules still face numerous challenges due to the biological and physiological obstacles of the GIT and the inherent characteristics of the drug itself (e.g., instability and large molecules) [48,120].

**Figure 1:**
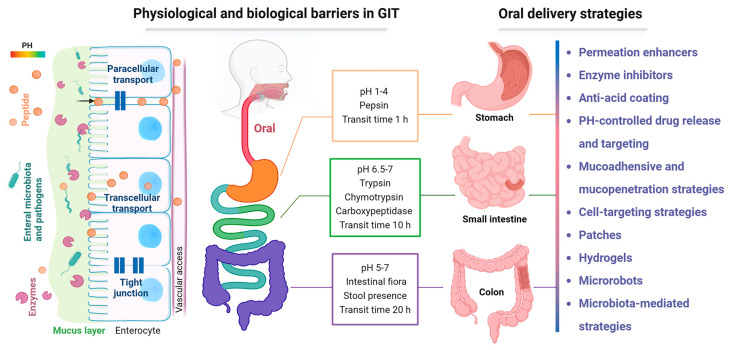
Barriers and strategies for oral delivery of biomacromolecules. Note: the strategies are not strictly region-specific. Created with Biorender.com.

Currently, only a very small number of oral dosage forms of biomacromolecular drugs are available on the market. For instance, oral semaglutide tablets are the only oral product of peptide GLP-1RAs approved by the FDA for the treatment of T2DM [38,121,122,123]. Oral semaglutide is a co-formulation of semaglutide with an absorption enhancer, sodium N-(8-[2-hydroxybenzoyl] amino) caprylate (SNAC), which locally neutralizes gastric acid and promotes the absorption of semaglutide in the gastric epithelium [115,116,124,125]. However, the oral bioavailability of oral semaglutide is still very low, approximately 0.4–1% [116,123,126,127]. Therefore, increasing the oral bioavailability of biologics remains the focus of current research and remains a major challenge for pharmaceutical industries. To improve the oral bioavailability of biomacromolecule drugs, strategies, including chemical modifications (e.g., cyclosporine cyclization and N-alkylation) [116,128], permeation enhancers (e.g., fatty acids and chitosan) [49,129], drug delivery systems (e.g., nanoparticles and microrobots) [50,113,119,130], targeted strategies (e.g., pH-sensitive materials and cell-targeting strategies) [38], and the combination of multiple strategies, are exploited [34,112]. In this section, we briefly introduce the biological and physiological barriers of the GIT, and we also review the oral delivery approaches for improving the oral bioavailability of biomacromolecules (Figure 1).

### 3.1. Absorption Barriers for Oral Delivery of Biomacromolecules

The complex environment of the GIT hinders the efficient uptake of biomacromolecules [128,130]. To achieve satisfactory bioavailability after oral administration, drug molecules must overcome several GI barriers, including biochemical barriers, mucosal barriers, and epithelial barriers [112,131]. To this end, understanding the physiology of the GIT and the absorption obstacles is important to the development of oral dosage forms, which determines the fate of biomacromolecules. The anatomy and physiology of the GIT have been thoroughly reviewed elsewhere in detail [34,38,50,112,114,120,128,132,133,134]. In this section, we only briefly describe the absorption barriers of biomacromolecules given orally (Figure 1).

Biochemical barriers with acidic pH and proteolytic enzymes such as proteases can lead to the degradation and inactivation of biomacromolecules before they enter the systemic circulation. Specifically, the pH of the GIT varies widely, from a lower stomach pH (1–2) to a higher gut pH (5–8). These large pH variations allow for the potential disruption of the integrity and stability of drug molecules, thereby affecting the GI absorption of drugs [34,135]. Various enzymes, including proteolytic and digestive enzymes, also affect the stability of drug molecules in the gut. Enzymatic degradation can occur at different sites of the GIT, such as the mucus layer [136,137], which varies in thickness throughout the GIT and serves as a protective barrier against pathogens and irritants [138,139] and also as a significant obstacle to the delivery of biomacromolecules [54,140]. Mucus is composed of mucins, glycoprotein, enzymes, and water, and it has a gel-like structure [51,114]. Mucin fibers are the main components of the mucus layer and are secreted by goblet cells [51,141]. Mucin fibers are glycoproteins rich in negatively charged glycosylation regions and hydrophobic domains. These fibers are entangled and crosslinked with each other through hydrophobic interactions [142], resulting in dense porous structures whose negatively charged and highly viscous properties can trap and impede the movement of particles and/or drugs. The GI epithelium, protected by the mucus layer, forms a physical barrier to the transport of drug molecules. The epithelial barrier consists of different intestinal cells and tight junctions (TJs). These compact structures only allow small hydrophilic molecules to pass through [120,133], while limiting the transcellular and paracellular transport of biomacromolecules. TJs act as a rate-limiting barrier for paracellular diffusion through the intestinal epithelium, as they restrict the passage of particles with sizes above 2 nm [141,143]. In addition, the specific functions of each cell type in the GI epithelium affect the absorption process [141,144].

### 3.2. Absorption Strategies for Oral Delivery of Biomacromolecules

Over the decades, with a detailed understanding of GIT biology and physiology, a wide array of advanced strategies for the oral delivery of biomacromolecules have emerged (Figure 1) [48,145]. These strategies work on a single barrier or multiple barriers to achieve effective bioavailability via the oral route. For example, nanocarriers, including lipid nanocarriers, polymer nanocarriers, lipid–polymer hybrid nanocarriers, and naturally derived nanocarriers show promise in oral delivery by improving the stability of encapsulated molecules [93,120,126,146]. The oral absorption of biomacromolecules can be further improved through surface modifications on the delivery carriers, such as adhesive polymer coating to improve the retention in the GIT [147], PEGylation to promote the penetration of the mucous layer [148,149], or ligand conjugation to further enhance the absorption of carriers/drugs in the GIT [150,151,152,153]. Herein in this section, we discuss the advanced oral delivery strategies for biomacromolecules based on the different gastrointestinal absorption barriers mentioned in the previous section (Figure 1).

#### 3.2.1. Acid–Base Balance and Enzymatic Inhibition

Alterations in the acid–base environment can lead to pH-induced oxidation, deamidation, or hydrolysis of biomacromolecules [117]. The presence of various proteolytic enzymes in the GIT, as well as the presence of sucrase and numerous peptidases located in different GI sites (e.g., the brush border membrane), contribute to the inactivation of biologic drugs [34]. Therefore, addressing the heightened sensitivity of biomacromolecule products to acidic and enzymatic degradation is crucial in the development of effective oral formulations.

Countermeasures include altering the formulation of the preparation, such as by converting a liquid preparation into a capsule or a tablet preparation or incorporating additional excipients that modify intestinal pH [154,155]. Octreotide extended-release capsules (Mycapssa) were approved by the FDA in 2020 [154]. They are prepared by suspending fine hydrophilic particles, including octreotide, C8, and polyvinylpyrrolidone, in an oil mixture to form a special oily suspension, which is then filled into gelatin capsules and coated with methacrylate (Acryl-EZE) [129]. Enteric coating is effective in preventing octreotide from being destroyed by gastric acid [156]. Hashim et al. developed an oral delivery platform, also known as RaniPill, which is an enteric-coated, capsule-like, and investigational device. RaniPill can deliver recombinant human insulin directly to the jejunal wall with a relative bioavailability close to 100%, comparable to subcutaneous injections [157]. Oral semaglutide is available on the market, in which SNAC is incorporated with semaglutide to improve the systemic absorption of the peptide by transiently neutralizing the acidic environment and inhibiting pepsin activity [158]. Organic acids can regulate the pH difference of 1–2 units in the GI environment, reducing the optimal pH value for proteolysis [155,159]. Calcium ions act as enzyme cofactors, and they can influence enzyme activity while simultaneously altering intestinal pH when citric acid (a calcium ion chelator) is added [155,160]. For example, the Phase III clinical trial of oral recombinant salmon calcitonin (Oracal) enhanced the stability of calcitonin in the stomach through an acid-resistant acrylic polymer (Eudragit L30 D55) and effectively improved the transport of calcitonin with the addition of citric acid to the tablet [161].

Enzyme inhibitors represent the most direct approach to solving the issue of enzymatic hydrolysis. ORMD-0801 [162], an oral insulin hypoglycemic capsule containing soybean trypsin inhibitor and permeation enhancers (ethylenediaminetetraacetic acid and bile salts) [38], has shown positive Phase II clinical results for the treatment of T2DM [163]. Furthermore, the ORMD-0801 used to treat metabolic dysfunction-associated steatohepatitis (MASH) was associated with a 6.9% reduction in magnetic resonance imaging–proton density fat fraction (indicating % liver fat) and a 23 dB reduction in mean fibrosis score (CAP/m) from baseline to week 12 (NCT02653300). These findings highlight the potential of enzyme inhibitors in the oral delivery of biomacromolecules.

In addition, the introduction of different amino acid sequences, spatial structures, or chemical structures can effectively increase the GI stability of biological macromolecular drugs. For instance, by chemically modifying the peptide structure or performing peptide cyclization, the exposure of the N-terminus and C-terminus of the peptide molecule can be minimized, thus enhancing resistance to enzymatic degradation. Trofinetide (Daybue) demonstrates peptide structure optimization [164], while Voclosporin (Lupkynis) exemplifies peptide cyclization [165]. Inclisiran, a siRNA drug that inhibits PCSK9 in hepatocytes, has chemical modifications, including the substitution of 2′-O-methylnucleotides or 2′-O-fluoronucleotides, which significantly enhance its stability [166]. Other strategies for protecting biological drugs include amino acid substitution (replacing natural amino acids), coupling, etc. [167,168].

Nanocarriers are used to protect biological drugs from acid destruction and enzyme degradation in the GIT and have achieved great success in extensive preclinical studies [130,136,169,170]. Appropriate formulations (e.g., lipids and polymers) enhance the capability of nanoparticles to overcome biochemical barriers in the GIT [132,171,172,173]. Self-emulsifying drug delivery systems protect the drug from enzymatic degradation and promote intestinal absorption by forming oil droplets in the GIT [174]. PH-responsive delivery systems facilitate the site-specific release of biomacromolecules within the GIT [175]. Of note, the nanocarriers can often be modified to further improve oral absorption (discussed in detail in the following sections).

#### 3.2.2. Mucoadhesion and Mucopenetration

The mucus layer is a natural physical and chemical barrier that traps pathogens and macromolecules, thereby limiting the invasion of foreign harmful substances [176,177], but it also presents a challenge to drug penetration [178,179]. Additionally, the diffusion and absorption of biomacromolecular drugs are also affected by the molecular structure, charge, and quantity of covalent molecules in the GI mucus layer [131]. To enhance the oral bioavailability of drugs, researchers are employing mucoadhesive and mucopenetrating biomaterials for the alternative surface modification of drug carriers.

Exploiting mucoadhesive polymers has become the most popular strategy for developing oral non-specific mucosal targeted delivery systems [149,152,178,180,181]. This strategy mainly uses modified polymer chains to entwine into the mucous network through hydrogen bonding, electrostatic forces, van der Waals forces, and hydrophobic interactions to extend the retention time in the mucus [172,176,178,182,183]. The electrostatic attraction between positively charged chitosan and negatively charged sialic acid and sulfate groups on mucin promotes strong adhesion [184,185]. Insulin-loaded poly (n-butylcyanoacrylate) nanoparticles coated with chitosan exhibited good stability and release profiles in the GIT [186]. Mumuni et al. developed an insulin-loaded nanoparticle that overcomes the mucus barrier with chitosan and water-soluble snail mucin as natural polymers, and it shows excellent hypoglycemic effects in diabetic rats after oral administration [187]. Sadio et al. utilized imidazole-modified chitosan nanoparticles to target caudal-related homeobox transcription factor 2 (CDX2), a regulator of gut differentiation, effectively delivering the siRNA locally to the stomach across the gastric mucus layer [188]. The mucous layer is rich in cysteine that contains active sulfhydryl groups, which can spontaneously react with other sulfhydryl groups to form disulfide bonds to strengthen the adsorption between drug carriers and mucus [189]. Thiolated polymers have been widely used, in which the introduction of immobilized thiol groups into mucoadhesive polymers can significantly improve adhesion [190,191,192]. For instance, the thiol group on the surface of preactivated thiolated chitosan nanoparticles forms disulfide bonds with the cysteine residues in the intestinal mucosa, extending the elimination of half-life by more than 4 times that of oral octreotide. In recent years, researchers have also found that grafting specific molecules, such as lectins, peptides, or bacterial invasions, onto the surface of drug carriers can not only enable mucus-binding but also increase the intestinal absorption of drugs through active targeting [152,190,193]. Zhang et al. designed lectin-modified solid lipid nanoparticles, which enhanced the stability of insulin in the GIT and increased the oral bioavailability of insulin to 7.11% [194]. Yin et al. prepared a core–shell nanosystem modified by the CSKSSDYQC peptide for goblet cell-targeting, where liraglutide eventually passed through the mucous layer [195].

Enhanced interaction with mucus is not the only parameter that improves the oral bioavailability of biomacromolecules. In addition to this, drug carriers still need to penetrate the mucus to reach the intestinal epithelium below. Mucus penetration strategies inspired by the behavior of viruses have been exploited [178]. Studies have shown that effective mucus penetration can be achieved when the drug carriers have a net-neutral, highly hydrophilic surface [178,190,196,197]. The dense coating of polyethylene glycol (PEG) on the surface of drug carriers has become a mainstream method in the engineering of mucus-penetrating drug delivery systems [148,149]. Yamazoe and her colleagues prepared PEGylated liposomes in which 10% PEGylated liposomes showed the best mucus permeability, allowing them to reach the bottom of the artificial mucus layer [148]. Other surface-modifying polymers, such as poly N-(2-hydroxypropyl) methacrylamide (pHPMA), have also been used to improve the mucus penetration of delivery systems [198]. Liu et al. found that pHPMA derivatives are assembled onto the surface of insulin-loaded trimethyl chitosan nanoparticles to form a hydrophilic polyanion coating [199]. The presence of the pHPMA coating enhances the diffusion of the nanoparticles in the mucus, preventing their trapping and rejection. This specialized polymer coating is designed to dissociate in the mucus, thus facilitating the diffusion of nanoparticles [198,200].

#### 3.2.3. Gastrointestinal Epithelial Absorption

The GI epithelium consists of different cell types, including epithelial cells, goblet cells, and M cells, as well as a complex array of molecular barriers, including TJs, adhesive junctions, and desmosomes [201]. To enhance the GI epithelial absorption, the most used strategies include transcellular transport and paracellular transport.

The transcellular pathway facilitates the transport of biomacromolecule drugs through the cytoplasm of intestinal cells and their subsequent absorption into the lymphatic or systemic circulation [170,202]. For transport via transcellular pathways, oral macromolecules must cross the epithelial cells directly into the vascular space adjacent to the basolateral surface of the intestinal epithelium [203]. Thus, transcellular PEs are also widely exploited [55]. SNAC, a marked PE, improves the transcellular transport of peptide GLP-1RAs by altering the fluidity of cell membranes [115,204]. Cell-penetrating peptides (CPPs) enable biological membranes to be traversed, even those affected by metabolic disorders. It has been indicated that CPPs could enhance the transcytosis of insulin across intestinal cells, thereby improving insulin absorption and therapeutic efficacy [205]. The incorporation of D-amino acids into CPPs can improve their stability and delivery efficiency [206]. Negatively charged anionic nanoparticles enhance intestinal epithelial permeability by binding to integrins, thereby facilitating the oral delivery of insulin or exenatide [207]. Increasing active targeting delivery in GI epithelium has also been widely exploited to increase the transcellular transport of biomacromolecules, such as M cell-targeting, dendritic cell (DC)-targeting, specific receptor- and transporter-targeting [170]. M cells are mainly located in Peyer’s patches [132]. Their unique structure facilitates the entry of specific substances into the submucosal lymphoid tissue through active transport pathways [152]. This can bypass first-pass metabolism and improve the bioavailability of the drug [170]. This approach is widely used in oral vaccines and has potential in protein drug delivery [151]. He et al. developed aptamer-modified liposomes by M cell-targeting for the oral delivery of exenatide, which reduced the transepithelial electrical resistance of M cells and increased the transport efficiency of exenatide by 2 times [153]. DC-targeting plays a crucial role in immune responses. Antigens and adjuvants are encapsulated in nanoparticles that bind to DC receptor-specific ligands (e.g., mannose receptors), which actively transport to the DCs, increasing antigen presentation and immune activation [208]. A variety of specific receptors and transporters are expressed on the surface of epithelial cells [150,170]. Nanoparticles modified by ligands, selectively bound to the receptors or transporters on epithelial cells, have the potential for biomacromolecule delivery. For example, 2,5-hydroxycholesterol (25HC)-modified nanoparticles have a high affinity for Niemann–Pick C1 Like 1 (NPC1L1) transporters on the apical side of the intestinal epithelium, enhancing endocytosis and stimulating basal ATP-binding cassette transporter A1 (ABCA1) to increase exocytosis, thereby improving the oral bioavailability of liraglutide [209]. Wu et al. prepared butyrate-functionalized nanoparticles that interact with monocarboxylate transporter-1 to enhance the cellular uptake and transepithelial transport of insulin after oral administration [210].

Paracellular transport allows biomacromolecules to enter the bloodstream for absorption by transiently disrupting cellular junctions among the enterocytes [55,211,212,213]. Paracellular PEs are one of the most commonly used and effective strategies, including acidity modifiers [55,214], chelating agents [124], surfactants [215,216], bile salts [217], cationic polymers [218], and some anionic nanoparticles [207]. Chelating agents (e.g., ethylenediamine tetra-acetic acid, EDTA) increase paracellular transport by depleting extracellular Ca2+, which forms the TJ and apical junction complexes [219]. EDTA, as a paracellular PE, significantly increases the mucosal permeability of enalaprilat and hexarelin in the rat intestine at concentrations of 5 mg/mL [220]. The surfactant decanoic acid (capric acid, C10) and octanoic acid (caprylic acid, C8) have both been used in oral formulations for biomacromolecule delivery [115,221]. C8 induces the recombination of TJ proteins such as ZO-1 and claudins, promoting paracellular transport [222,223]. Tran et al. developed C10 and SNAC erodible tablets for the gastric delivery of a GIP/GLP1 peptide (LY) in monkeys. The LY bioavailability of C10 and SNAC tablets was similar (5.7% and 4.2%, respectively). Chitosan also improves the paracellular transport of biomacromolecules by opening TJs [224,225]. Yang et al. encapsulated exenatide into chitosan nanoparticles and then coated sodium alginate, which enhanced the oral absorption of exenatide and had a significant hypoglycemic effect in vivo [226]. Recently, anionic nanoparticles, such as silica nanoparticles, have been found to induce TJ relaxation by binding to integrins, thereby increasing intestinal permeability and facilitating the oral delivery of peptides and proteins, such as exenatide and insulin [207].

#### 3.2.4. Gastrointestinal Epithelial Injection

Over the last decade, oral microrobots have emerged and shown great promise in the oral delivery of biomacromolecules [58]. Oral microrobots are small devices with microneedle injectors that transcend the diffusion process of traditional strategies, and some even achieve an oral absorption efficiency consistent with the traditional injection of macromolecular drugs. Traverso et al. have developed a luminal unfolding microneedle injector (LUMI) for the oral delivery of macromolecule drugs. LUMIs safely and effectively deliver insulin to the small intestine in swine. Compared with subcutaneous injections, the effect of LUMIs is faster, and their systemic absorption is greater than 10% within 4 h [227]. From 2019 to 2024, oral microrobots were introduced almost every year, achieving initial success in large animal experiments and presenting new opportunities in the oral delivery of biomacromolecules. However, there is limited public information on the specific progress of oral microrobots from animal trials to clinical trials. Oral microrobots still face multiple challenges, such as adverse reactions (e.g., GI wall damage) and tolerability [58].

## 4. Application of Oral Biomacromolecules for Metabolic Diseases

Metabolic diseases are characterized by metabolic disorders in organs, tissues, or cells caused by abnormal synthesis and the decomposition of glucose, lipids, proteins, and other substances [228,229]. Common metabolic diseases, such as diabetes, obesity, hypercholesterolemia, and MASLD, have become a global health burden over the last few decades [5,230]. Diabetes is the most prevalent metabolic disease characterized by elevated levels of blood glucose due to an impaired ability of the body to produce or respond to insulin [231,232], and it primarily includes type 1 diabetes mellitus (T1DM) and T2DM [233]. Complications associated with diabetes include cardiovascular disease, nephropathy, retinopathy, and neuropathy [234,235,236]. Hypercholesterolemia, also known as high cholesterol and hyperlipidemia, is a condition in which too many lipids accumulate in the blood [237,238]. MASLD, tightly linked to patients with diabetes, obesity, hypercholesterolemia, and hypertension, is defined as the presence of excess lipids in the liver [239]. MASLD is associated with an increased risk of cirrhosis and hepatic and extrahepatic carcinomas [240]. Obesity and overweight are defined as an abnormal energy balance that increases the risk of diabetes, hypertension, coronary heart disease, stroke, and other serious health issues [241]. It is noteworthy that oral biomacromolecules play an important role in alleviating abnormal metabolism in the body. In this regard, we discuss the strategies under which oral administration of biomacromolecules alleviates metabolic defects.

### 4.1. Regulation of Glucose Metabolism

Glucose metabolism is a biochemical process involving the absorption, transformation, synthesis, degradation, and utilization of glycans [242,243]. Insulin and glucagon work together to maintain normal glucose levels in the body, where insulin binds to the insulin receptor and facilitates the translocation of the glucose transporter from intracellular vesicles to the surface of the cell membrane, thereby promoting glucose uptake and utilization [244,245]. GLP-1 is a gut hormone secreted by enteroendocrine L cells with multiple physiological functions, such as stimulating insulin secretion and inhibiting glucagon release [246]. Insulin and GLP-1RAs are the most widely used biomacromolecular drugs for hypoglycemia [247]. Oral insulin mimics the endogenous insulin secretion process in the body, enabling the liver to regulate glucose in time [119]. This approach effectively alleviates the metabolic dysfunction associated with non-endogenous insulin and represents a unique advantage of oral administration [38]. Oral administration of GLP-1RAs has similar physiological advantages, which can simulate the intestinal secretion pathway of native GLP-1 [38,119]. Although oral insulin and GLP-1RAs remain one of the great challenges facing the pharmaceutical industry, it is considered a safer and more convenient method for the management of hypoglycemia.

#### 4.1.1. Insulin

Several oral insulin formulations with different strategies have entered clinical trials to enhance bioavailability. IN-105 (Tregopil), developed by Biocon, modifies insulin molecule PEG side chains at B29 for increasing solubility and stability and adds the PE sodium caprate to the insulin Tregopil for facilitating rapid absorption [248,249,250]. ORMD-0801 (developed by Oramed) encapsulates insulin in a special capsule with acid-resistant coating and protease inhibitors and is a supplement to enhance adsorption [163]. Emisphere’s oral insulin candidate uses the carrier molecule monosodium N-(4-chlorosalicyloyl)-4-aminobutyrate (4-CNAB) to interact with insulin and be better absorbed by the GIT [38,251]. Capsulin by Diabetology is an enteric-coated capsule that protects insulin from gastric degradation and enhances adsorption outside the intestinal wall [252]. Nodlin is developed with NOD technology, containing insulin nanoparticles in a bioadhesive enteric-coated capsule to improve retention and absorption in the GIT [253]. Each strategy has its own limitations, such as the need to address long-term safety and efficacy in diverse patient populations. Very few oral insulin formulations have entered Phase III clinical trials. Oral insulin ORMD-0801 was approved for Phase III clinical trials in 2014. However, the latest trial results indicate that ORMD-0801 did not significantly reduce glycated hemoglobin (HbA1c) levels, a key efficacy indicator for evaluating the long-term effects of diabetes treatment (NCT04606576, NCT04754334). This outcome suggests that ORMD-0801 did not meet regulatory standards for drug approval [38]. In contrast to the setbacks experienced by ORMD-0801, Tregopil successfully completed a Phase III study in 2020 (NCT03430856). Tregopil demonstrates rapid action and exhibits a favorable safety profile, which effectively increases blood glucose levels 1 h after meals and improves overall postprandial blood glucose control [250]. Currently, it is the oral insulin with the greatest potential for clinical approval. However, to date, no oral insulin has been approved for clinical use [143].

**Figure 2:**
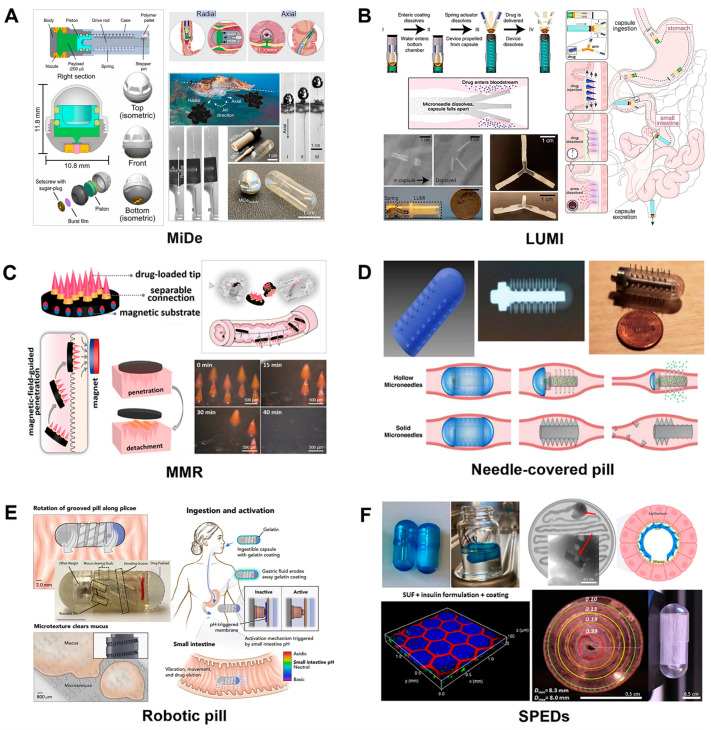
Intelligent microrobot pill delivery systems used for oral insulin against hyperglycemia. (**A**) The microjet delivery (MiDe) systems. (**B**) The luminal unfolding microneedle injection (LUMI) device. (**C**) The magneto-responsive microneedle robots (MMRs). (**D**) The cylindrical needle-covered pill. (**E**) The robotic mucus-clearing capsule (RoboCap). (**F**) The self-unfolding proximity enabling devices (SPEDs). Adapted and reproduced with permission from ref. [135,227,255,261,262,263,264].

Recent advances in biomaterials and nanotechnology have led to the emergence of innovative drug delivery systems [119], such as PEs [129], nanoparticles [50,113,119,130], and oral microrobots [58], which show considerable promise in glucose metabolism by the oral delivery of anti-diabetic biomacromolecules (such as insulin) in numerous preclinical studies [119,254,255,256,257,258,259]. In 2019, the emergence of a self-orienting millipost array (SOMA) triggered a strong enthusiasm for the development of oral microrobotics. SOMAs, inspired by the leopard tortoise, can self-reorient from any starting position to attach to the gastric wall, which allows the trigger of the millions of insulin-loaded carriers to the gastric mucosa. SOMAs are safe and can deliver insulin with plasma levels comparable to subcutaneous administration [260]. The safety of an unloaded SOMA has been demonstrated in a human trial (NCT05314283). In recent years, several intelligent microrobots have been developed for oral insulin delivery, including microjet delivery (MiDe) systems [261], LUMIs [227], magneto-responsive microneedle robots (MMRs) [262], cylindrical needle-covered pills [263], robotic mucus-clearing capsules (RoboCaps) [264], and self-unfolding proximity-enabling devices (SPEDs) [255] (Figure 2).

#### 4.1.2. Peptide Glucagon-like Peptide-1 Receptor Agonists

As we mentioned earlier, oral semaglutide is only an oral GLP-1RA formulation on the market for the treatment of T2DM, with limited oral bioavailability [43,44,127,265,266]. To enhance the efficiency of oral GLP-1RAs in controlling blood glucose levels, a number of strategies are exploited, which have shown promising results in rodent and/or primate models. Araujo et al. developed a multifunctional composite oral system that utilizes droplet microfluidic technology for the oral dual delivery of GLP-1 analogue and dipeptidyl peptidase 4 inhibitors. This multifunctional composite oral system enhanced the hypoglycemic effects in a T2DM rat model, reducing blood glucose levels by 44% after 4 h [267]. Exosomes have shown significant promise as vehicles for the oral delivery of biomacromolecules due to their superior capacity to traverse epithelial barriers [268]. Xiao et al. developed a liposome–milk exosome hybrid vesicle with self-adaptive surface properties for the oral administration of semaglutide. The authors introduced a pH-sensitive hydrazone bond between the highly hydrophilic zwitterionic polymer and the phospholipid, taking advantage of the pH microenvironment on the jejunum surface, and achieved oral bioavailability of up to 8.7% [269]. Fc receptor (FcRn) is highly expressed on the luminal surface of the epithelial cells and plays a key role in mediating transcellular transport [270]. FcRn-targeted nanoparticles loaded with semaglutide after one week of daily oral administration showed improved glycemic regulation in T2DM mice compared to oral free semaglutide or non-targeted nanoparticles [265]. In recent years, the activation of GLP-1 receptors through oral nucleic acid delivery has also attracted extensive interest as a long-acting strategy against hyperglycemia with a reduced frequency of administration [271,272]. The apical sodium-–bile acid transporter (ASBT) pathway has proven to be an attractive target for oral biomacromolecules [273,274]. Shahriar et al. reported a multimodal carrier system, with the excipients of bile salt, protamine sulfate, and calcium phosphate, for the oral delivery of a plasmid-encoding GLP-1. The system could protect the plasmid in the GIT and transport it to the target site via ASBTs, effectively improving insulin sensitivity and maintaining glucose levels at a normal state for a prolonged period in diabetic mice by a single-dose administration [275]. In recent years, oral microrobots have been used to improve the oral absorption of peptide GLP-1RAs in alleviating hyperglycemia. Traverso et al. designed a dynamic omnidirectional adhesive microneedle system (DOAMS), which had a biomimetic core–shell structure and showed strong dynamic adhesion properties in non-human primates. Compared with traditional tablets, DOAMS-modified tablets significantly enhance the oral absorption of semaglutide, and the area under the plasma concentration–time curve is improved two-fold [276]. In addition, after delivering 4 mg of inactivated semaglutide-like GLP-1 analogue via the liquid-injecting self-orienting millimeter-scale applicator (L-SOMA), drug exposure could be detected in the plasma within 15 min. This bioavailability could reach 103 ± 42%, while it was 78 ± 4% for subcutaneous injections. These data indicate that oral microrobots can effectively deliver GLP-1 analogue, playing better roles in regulating blood glucose, promoting insulin secretion, and inhibiting glucagon secretion [277].

### 4.2. Regulation of Lipid Metabolism

Lipid metabolism involves the synthesis and degradation of fatty acids and/or more complex lipid molecules (e.g., phospholipids, cholesterol, and glycolipids) [278,279]. Abnormal lipid profiles (dyslipidemia) have been associated with a range of metabolic diseases, such as metabolic syndrome, CDVs, T2DM, and MASLD [4,280,281,282,283].

#### 4.2.1. Proprotein Convertase Subtilisin/Kexin Type 9 Inhibitors

**Figure 3:**
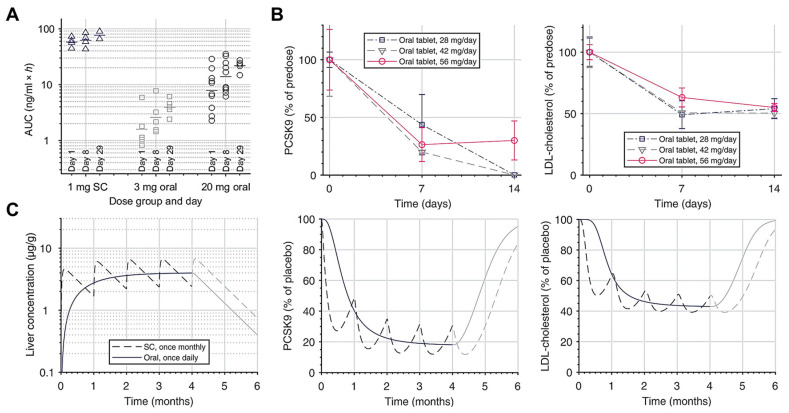
Pharmacokinetic (PK) and pharmacodynamic (PD) evaluations of AZD8233 tablets in dogs and cynomolgus monkeys and predicted PKPD parameters in humans. (**A**) The area under curve (AUC) of repeated daily oral administrations of AZD8233 tablets in dogs. (**B**) The inhibition percentage of PCSK9 and LDL cholesterol in healthy cynomolgus monkeys after AZD8233 administrations by the oral and subcutaneous routes, respectively. (**C**) Predicted PKPD parameters of AZD8233 in humans by daily oral administration versus monthly subcutaneous administration. Adapted and reproduced with permission from ref. [287].

PCSK9 was identified as a highly promising therapeutic target for the prevention and treatment of atherosclerotic CDV [284,285,286]. PCSK9 inhibitors are effective therapeutics to treat dyslipidemia, by reducing the LDL-C [280]. The mechanism of action is to inhibit PCSK9 to promote LDL receptor (LDL-R) recycling and enhance the uptake of LDL-C by liver cells, thereby reducing the plasma levels of lipoproteins [287,288]. Currently available PCSK9 inhibitors, including PCSK9 monoclonal antibodies and PCSK9 siRNA, are all administered by injection [289,290]. Despite nearly 20 years of effort, oral PCSK9 inhibitors have yet to be developed, though the enthusiasm for research in this field has not diminished [286]. Recent advancements in oral PCSK9 inhibitors have demonstrated promising efficacy in addressing lipid metabolism and hypercholesterolemia in clinical trials or preclinical studies [291,292]. MK-0616 is a macrocyclic peptide with exquisite potency and selectivity for the LDL-R-binding domain of PCSK9 [293]. The oral bioavailability of MK-0616 exhibited a satisfactory bioavailability of approximately 2% when a permeation enhancer sodium caprate was added to the formulation [286,293]. Phase I clinical trials have shown that a single oral dose of MK-0616 can inhibit the PCSK9 for more than 24 h [293]. Multiple-oral-dose regimens achieved a reduction in LDL-C levels of up to about 61% in patients after 14 days of one treatment per day [293]. Phase II clinical data further supports its efficacy and safety in patients with atherosclerotic CVD [292]. Given these obtained clinical data, the cyclic peptide MK-0616 may provide an alternative for patients who are intolerant to statins or who have failed existing therapies, and oral lipid-lowering agents may improve adherence and treatment outcomes. Large-scale Phase III trials evaluating long-term benefits and safety are ongoing to confirm whether it can be used as an option for lipid management and cardiovascular risk reduction (NCT05952856) (NCT05952869). Besides oral cyclic peptides, oral PCSK9 inhibitors that are currently being developed also include antisense oligonucleotides (ASOs). AZD8233, also called ION-863633, is a highly potent N-acetylgalactosamine (GalNAc) ASO targeting PCSK9 mRNA. In a Phase IIb trial (NCT04641299), a single subcutaneous dose of 90 mg AZD8233 effectively reduced PCSK9 by more than 90%, with a maximum average reduction of nearly 70% in LDL-C levels in patients with dyslipidemia. The feasibility of the oral administration of AZD8233 has also been demonstrated and showed a bioavailability of 7% in the targeted organ (liver) (Figure 3) [287]. Specifically, PCSK9 ASO was co-formulated in a tablet with sodium caprate. Daily oral tablets with 700 mg of sodium caprate had a liver bioavailability of 7% in dogs. Its efficiency was further confirmed in cynomolgus monkeys, in which plasma PCSK9 and LDL-C levels were reduced after oral administration. Assuming 5% liver bioavailability in humans, an oral dosage of 15 mg per day is expected to reduce PCSK9 by 80% in a steady state, which supports the development of an oral dosage form of PCSK9 ASO for the treatment of dyslipidemia.

#### 4.2.2. Peptide Glucagon-like Peptide-1 Receptor Agonists

GLP-1 can regulate lipid metabolism through its actions on various organs such as the liver, pancreas, and adipose tissue, thereby promoting a more favorable lipid profile [294,295]. The excessive secretion of the apolipoprotein B48 (ApoB48) is the main cause of postprandial dyslipidemia [296,297]. Clinical trials have shown that GLP-1RAs, such as exenatide (NCT0105654, NCT00097500), liraglutide (NCT02721888), lixisenatide (NCT02049034), and semaglutide (NCT02079870), can reduce postprandial lipids and lower ApoB-48 levels. GLP-1 also inhibits lipolysis in adipose tissue, thereby reducing circulating free fatty acids, which may help improve the blood lipid profile and reduce the risk of diabetes in patients with CVDs [296,298,299,300]. A randomized, double-blind, crossover trial evaluated the effect of oral semaglutide (Rybelsus) on lipid metabolism in patients with T2DM (NCT02773381). After 12 weeks of treatment, Rybelsus reduced triglycerides, LDL, and ApoB48 compared with the placebo [301]. Triglyceride and LDL levels are associated with the progression of MASLD. Several advanced strategies have investigated the efficiency of GLP-1RAs for MASLD management via the oral route [302,303]. It has been reported that encapsulating peptide GLP-1RAs (e.g., exenatide and semaglutide) in reverse micelle-loaded lipid nanocapsules could help alleviate the pathological changes associated with MASLD [304,305]. In these studies, although there was no significant change in lipid accumulation in the liver after the long-term oral administration of GLP-1RA-loaded lipid nanocapsules, a reduction in relevant inflammatory factors was observed [304].

#### 4.2.3. Fibroblast Growth Factor 21

FGF21, a class of hepatokines that regulate lipid and glucose metabolism, has garnered much attention due to its translational potential for the management of obesity-related metabolic comorbidities [306,307,308]. The FGF21 signaling pathway requires co-receptor β-klotho (KLB) to work with the FGF receptor to have pleiotropic metabolic effects, including the induction of fatty acid oxidation and ketogenesis in the liver [309]. FGF21 and tissue-specific accessory receptor KLB are highly expressed in the liver [310], which makes the pharmacological effects of FGF21 more interesting in improving lipid metabolism in the liver. Numerous clinical trials have been registered to evaluate the efficacy of FGF21 analogues in the treatment of obesity, T2DM, hypertriglyceridemia, and MASH [311,312]. In these trials, the primary endpoint of glycemic control was not met, while substantial improvements were observed in patients with MASH in dyslipidemia, liver fat content, and serum markers of liver fibrosis [313,314,315,316]. The FGF21 analogues under development are all injected. FGF21 has the potential to reverse this liver dysfunction, but it is essential to achieve targeted delivery to the liver, which can be facilitated by oral administration. However, since the FGF21 analogues are all in the developmental stage, the development of their oral dosage forms is also at a very early stage. To date, only one preclinical study has shown that the oral bioavailability of FGF21 analogues can be improved. Li et al. used milk-derived exosomes to encapsulate FGF21 and further incorporated transferrin on the surface of delivery systems, thus promoting FGF21 analogue transport into the systemic circulation via the oral route [317]. It has been demonstrated that the combination of FGF21 with other metabolic regulators, such as GLP-1, could enhance therapeutic effects [318]. Dual-agonist (GLP-1/FGF21) formulations injected once a week have demonstrated superior efficacy in anti-MASH compared to monotherapy [318,319]. However, to date, no oral formulation of FGF21-based multi-agonists has been reported.

### 4.3. Regulation of Energy Metabolism

Energy metabolism involves multiple and complex mechanisms that regulate energy intake and expenditure [320]. To maintain a stable weight, energy intake must be exactly equal to energy expenditure over time, a state known as energy balance [321]. Overweight occurs when energy intake exceeds energy expenditure. Several gut hormonal-based pharmacotherapies targeting energy expenditure are currently being explored and developed [322,323,324].

#### 4.3.1. Incretin-Based Biotherapies

Incretin hormones, GLP-1, and GIP are responsible not only for regulating glucose and lipid metabolism but also for affecting energy balance. The central nervous system is important for the maintenance of energy balance [325]. Both GLP-1 and GIP receptors are expressed in key feeding centers of the brain (e.g., hypothalamus and hindbrain), and the activation of these receptors is involved in the central regulation of energy balance [325,326,327]. Incretin hormone-based therapy is becoming a novel strategy for the medical management of overweight [328,329].

Given the physiological effects of GLP-1, not only on the metabolic disturbances of glucose but also on appetite and food intake related to energy balance in the body, the peptide GLP-1RA treatment is considered to be an effective class of weight loss drugs [330,331]. Clinical studies specifically registered for weight management have shown that oral semaglutide tablets (Rybelsus) can significantly reduce weight in obese patients, with participants losing approximately 9.6% of their baseline body weight [330]. MEDI7219 is a bis-lipidated peptide GLP-1RA that is specifically intended for oral administration. The bioavailability of its oral enteric-coated tablets can reach ~6% in dogs when combined with the PEs of sodium chenodeoxycholate and propyl gallate [332]. Over a 14-day period, dogs who took 10 mg MEDI7219 oral tablets once a day lost significantly more weight than those who took a placebo [332]. AMP-Sema, a colon delivery system based on organometallic phyllosilicate, has been developed for oral semaglutide. In diabetic rats, oral EBP-SEMA (equivalent to 8 mg/kg semaglutide) significantly reduced body weight compared to free drugs [333].

GIP is involved in the regulation of energy balance, although its exact role in weight maintenance is unclear [326,327]. Currently, no GIP agonist or antagonist monotherapies have been approved for marketing, but in recent clinical trials, GIP/GLP-1 receptor co-agonists, such as tirzepatide, have shown greater weight loss, even better than long-acting GLP-1RA alone [334]. The FDA approved tirzepatide injections (Zepbound) for chronic weight management in adults who are obese or overweight with at least one weight-related disease (e.g., T2DM, dyslipidemia, and hypertension) in November 2023 [335]. At present, there are no reports on the development of oral dosage forms of GIP/GLP-1 receptor co-agonists.

#### 4.3.2. Peptide YY

Peptide YY (PYY) is a hormone released by enteroendocrine L cells, which can work synergistically with other hormones regulating hunger and satiety, such as leptin, affecting appetite and energy intake [336]. Notably, the PYY3-36 form, in particular, has been shown to reduce food intake by activating the neuropeptide Y2 receptor (Y2R) in the hypothalamus, thereby contributing to weight management and metabolic homeostasis [337,338]. Vitamin B12 (B12) can cross the blood–brain barrier via a transcobalamin II-mediated pathway [339]. Some studies have explored coupling strategies, such as conjugating PYY3-36 to B12 (PYY3-36-B12), which has similar agonist activity at the Y2R, and has been observed in vivo with improved food intake inhibition [340]. Of concern, oral PYY3-36-B12 conjugates resulted in a substantial increase in plasma PYY3-36 concentration in rats (a nearly 18-fold increase compared to oral free PYY3-36) [341], which may achieve weight loss results associated with clinical injections. He et al. developed pH-responsive chitosan nanocarriers for the oral delivery of PYY, which crosslinked with toluene diisocyanate [342]. Kalomoiri et al. synthesized 16 novel neo-glycolipids and found that the use of glycolipids as PEs can enhance the penetration of PYY3-36 while maintaining the integrity of the cell monolayer, thus improving the oral absorption of PYY3-36 [165]. All these studies only investigate the behaviors of developed delivery systems in vitro. Clinical studies on oral PYY dosage forms are also rare. An early clinical trial showed that PYY3-36, when formulated with a PE (SNAC), can be rapidly absorbed from the intestine [343]. The oral administration of GLP-1 and PYY3-36 had a significant effect on appetite, showing a reduction in energy intake [343].

## 5. Conclusions and Perspective

Biomacromolecule therapeutics are crucial for metabolic diseases but face challenges in oral delivery due to gastrointestinal barriers. Although there are only a few oral biologics available clinically, continued research in this area is essential. Recent advances in biotechnology and nanotechnology have permitted some promising strategies for the oral delivery of biomacromolecules, such as PEs, nanoparticulate systems, and oral microrobots. PEs are one of the most used technologies. At present, the oral dosage forms of biomacromolecules that have been marketed or are undergoing clinical trials are mainly based on the PE strategy. However, the use of PEs is often limited by potential mucosal irritation and inconsistencies in absorption. Nanoparticles have developed rapidly in the past few decades. Despite the great success of this strategy in preclinical trials, there are currently no related products on the market due to the complexity and safety of the delivery method. Although oral microrobots were introduced late, most of their results have been verified on large animals, showing a good transformation prospect. Nevertheless, high production costs, potential GI wall damage, and regulatory hurdles remain significant barriers to their widespread clinical use. In the future, we look forward to significant advances in oral biomacromolecular therapies for metabolic diseases.

## Figures and Tables

**Table 1:** FDA-approved biomacromolecule drugs for the treatment of metabolic diseases.

Drug Ingredient	Trade Name	FDA-Approved	Indication	Routes	Targeting	References
Insulin	Iletin *	1923	Diabetes mellitus	s.c.	Insulin receptor	[75,79]
	Humulin R	1982		s.c.		
	Humulin N	1982		s.c.		
	Humulin L	1985		s.c.		
	Humulin BR	1986		s.c.		
	Humulin U	1987		s.c.		
	Novolin L	1991		s.c.		
	Novolin R	1991		s.c.		
	Velosulin BR	1999		s.c.		
	Exubera	2006		inh		
	Afrezza	2014		inh		
	Myxredlin	2019		i.v.		
Insulin lispro (fast-acting insulin analogue)	Humalog	1996	Diabetes mellitus	s.c.	Insulin receptor	[75,80]
	Admelog	2017		s.c.		
	Lyumjev	2020		s.c. or i.v.		
Insulin aspart (fast-acting insulin analogue)	NovoLog	2000	Diabetes mellitus	s.c.	Insulin receptor	[75,81]
	Fiasp	2017		s.c.		
Insulin glulisine (fast-acting insulin analogue)	Apidra	2004	Diabetes mellitus	s.c.	Insulin receptor	[75,82]
Insulin neutral protamine Hagedorn (NPH) (intermediate-acting insulin analogue)	Novolin N	1991	Diabetes mellitus	s.c.	Insulin receptor	[75]
Insulin glargine (long-acting insulin analogue)	Lantus	2000	Diabetes mellitus	s.c.	Insulin receptor	[83,84,85]
	Toujeo	2015				
	Basaglar	2015				
	Semglee	2021				
	Rezvoglar	2021				
Insulin detemir (long-acting insulin analogue)	Levemir	2005	Diabetes mellitus	s.c.	Insulin receptor	[75]
Insulin degludec (ultra-long-acting insulin analogue)	Tresiba	2015	Diabetes mellitus	s.c.	Insulin receptor	[75]
Insulin mix (NPH/regular; lispro protamine/lispro; aspart protamine/aspart; degludec/aspart; glargine/lixisenatide)	Humulin 70/30	1989	Diabetes mellitus	s.c.	Insulin receptor	[86,87,88,89,90]
	Novolin 70/30	1991		s.c.		
	Humulin 50/50	1992		s.c.		
	Humalog 50/50	1999		s.c.		
	Humalog 75/25	1999		s.c.		
	Novolog 70/30	2001		s.c.		
	Ryzodeg 70/30	2015		s.c.		
	Xultophy 100/3.6	2016		s.c.		
	Soliquo 100/33	2016		s.c.		
Insulin-like growth factors	Increlex	2005	Primary insulin-like growth factor-1 deficiency	s.c.	Insulin receptor	[91]
Exenatide	Byetta	2005	T2DM	s.c.	GLP-1 receptor	[92]
Exenatide PLAG NPs	Bydureon	2017	T2DM	s.c.	GLP-1 receptor	[93]
Liraglutide	Victoza	2010	T2DM	s.c.	GLP-1 receptor	[94,95]
	Saxenda	2014	Obesity	s.c.		
Lixisenatide	Adlyxin ^#^	2013	T2DM	s.c.	GLP-1 receptor	[96]
Albiglutide	Tanzeum ^$^	2014	T2DM	s.c.	GLP-1 receptor	[97]
Dulaglutide	Trulicity	2014	T2DM	s.c.	GLP-1 receptor	[98]
Semaglutide	Ozempic	2017	T2DM	s.c.	GLP-1 receptor	[99,100,101]
	Rybelsus	2019	T2DM	p.o.		
	Wegovy	2021	Obesity	s.c.		
Tirzepatide	Mounjaro	2022	T2DM	s.c.	GLP-1 and GIP receptors	[63,102]
	Zepbound	2023	Obesity	s.c.	GLP-1 and GIP receptors	[64,103]
Alirocumab	Praluent	2015	Hyperlipidemia; prevention of cardiovascular events	s.c.	PCSK9	[104]
Evolocumab	Repatha	2015	Hyperlipidemia; prevention of cardiovascular events	s.c.	PCSK9	[105,106]
Inclisiran (PCSK9 siRNA)	Leqvio	2021	Hyperlipidemia or mixed dyslipidemia in adults	s.c.	PCSK9	[72,76]
Sincalide	Kinevac	1976	Gallbladder function test, stimulate gallbladder contraction and pancreatic secretion	s.c.	Cholecystokinin-A (CCK-A) receptor	[74]
Sermorelin	Geref	1991	Growth hormone deficiency	s.c.	GHRH receptor	[74]
Tesamorelin	Egrifta	2010	Reduce abdominal fat in HIV-lipodystrophy	s.c.	GHRH receptor	[107]
Glucagon	Baqsimi	1998	Severe hypoglycemia	i.v., i.m, or s.c.	Glucagon receptor	[74]
Pramlintide	Symlin	2005	Glycemia control	s.c.	Amylin receptors	[74,108]
Metreleptin	Myalept	2014	Lipodystrophy	s.c.	Leptin receptor	[109]
Givosiran (siRNA)	Givlaari	2019	Acute hepatic porphyria	s.c.	ALAS 1	[76]
Setmelanotide	Imcivree	2020	Severe obesity caused by genetic disorders	s.c.	MC4 receptor	[110]
Evinacumab-dgnb	Evkeeza	2021	Homozygous familial hypercholesterolemia	i.v.	ANGPTL3	[77,78]

Abbreviations: s.c., subcutaneous; i.m., intramuscular; i.v., intravenous; p.o., oral administration; inh, inhalation. ALAS 1, 5-aminolevulinate synthase 1; ANGPTL3, angiopoietin-like 3; GHRH, growth-hormone-releasing hormone; IGF-1, insulin-like growth factor-1; GLP-1, glucagon-like peptide-1; GIP, glucose-dependent insulinotropic polypeptide; MC1R, melanocortin 1 receptor; MC4R, melanocortin 4 receptor; PCSK9, proprotein convertase subtilisin/kexin type 9. * Iletin ended under the Food and Drug Administration Modernization Act of 1997. $ Albiglutide was discontinued in 2017 due to limited prescribing. # Lixisenatide was discontinued in 2023 due to business considerations.

## Data Availability

No new data was created in this review.
